# Polyphosphate recovery by a native *Bacillus cereus* strain as a direct effect of glyphosate uptake

**DOI:** 10.1038/s41396-019-0366-3

**Published:** 2019-02-11

**Authors:** Alejandra Guadalupe Acosta-Cortés, Cesar Martinez-Ledezma, Ulrico Javier López-Chuken, Garima Kaushik, Surendra Nimesh, Juan Francisco Villarreal-Chiu

**Affiliations:** 1 Universidad Autónoma de Nuevo León, Facultad de Ciencias Químicas, Laboratorio de Biotecnología. Av, Universidad S/N Ciudad Universitaria, San Nicolás de los Garza, Nuevo León 66455 Mexico; 2 Universidad Autónoma de Nuevo León, Facultad de Ciencias Químicas, Laboratorio de Investigación en Ciencias Ambientales. Av, Universidad S/N Ciudad Universitaria, San Nicolás de los Garza, Nuevo León 66455 Mexico; 3 Department of Environmental Science. School of Earth Science, Central University of Rajasthan, Ajmer, Rajasthan 305817 India; 4 Department of Biotechnology. School of Life Sciences, Central University of Rajasthan, Ajmer, Rajasthan 305817 India

**Keywords:** Bacteria, Environmental microbiology, Applied microbiology

## Abstract

Seven bacterial strains isolated from a glyphosate-exposed orange plantation site were exposed to 1 mM N-(phosphonomethyl)glycine supplied as a phosphorus source. While some exhibited good biodegradation profiles, the strain 6 P, identified as *Bacillus cereus*, was the only strain capable of releasing inorganic phosphate to the culture supernatant, while accumulating polyphosphate intracellularly along the experimentation time. The composition and purity of the intracellular polyphosphate accumulated by the strain 6 P were confirmed by FTIR analysis. To date, the biological conversion of glyphosate into polyphosphate has not been reported. However, given the importance of this biopolymer in the survival of microorganisms, it can be expected that this process could represent an important ecological advantage for the adaptation of this strain to an ecological niche exposed to this herbicide. The polyphosphate production yield was calculated as 4 mg l^−1^, while the glyphosate biodegradation kinetic constant was calculated on 0.003 h^−1^ using the modified Hockey–Stick first-order kinetic model, with a half-life of 279 h. Our results suggest that *B. cereus* 6 P is a potential candidate for the generation of an innovative biotechnological process to produce polyphosphate through the biodegradation of the herbicide glyphosate.

## Introduction

Society has long depended on fertilizers for the improvement of crop productivity [1]. From manure and human excreta to guano and phosphate rock, the use of phosphorus (P) to increase crop yields has become inevitable for coping with the exponential growth of human population [2]. In 2009, more than 21 Mt of elemental P was extracted from phosphate rock, of which almost 18 Mt were used as fertilizers [3]. Despite its effectivity in promoting plant growth [4], nearly 100% of P consumed by animals and humans is excreted [5], leading to an increasing input of P across urban and rural land-water interfaces [6]. The resulting excess of nutrients in the environment is directly responsible for the extraordinary growth of algae that generate the eutrophication of surface water bodies worldwide [7]. It has been estimated that P recovery from wastewaters would not only avoid eutrophication in surface waters and coastal zones, but it would also offer a renewable source of P that could benefit the predicted phosphate rock scarcity in the near future [8].

Nowadays, the recovery of P from wastewater is usually attained by the chemical precipitation of struvite [9] or hydroxyapatite [10]. It can also be obtained by the production of P-rich sludges by a microbial process known as enhanced biological phosphorus removal (EBPR) [11]. The P-rich products of such methods can be directly used as plant fertilizers with the same effectivity as mineral phosphate [12], making these processes economically attractive [13]. Moreover, purified P in the form of polyphosphate (polyP) can be obtained directly from EBPR biosolids by biological [14] or thermal-chemical methods [15]. This biopolymer can be furtherly used in the synthesis of anticorrosion pigments [16, 17], fish processing [18, 19], and water treatment [20]. So, as global reserves of high-quality phosphate rock are becoming limited and its use by industries continues proliferating, the development of new strategies for recycling P becomes an important issue for future food security [21].

Despite the successful implementation of EBPR in wastewater treatment plants around the world, some forms of organophosphorus compounds, including organophosphate pesticides, are not entirely metabolized by polyphosphate-accumulating organisms. Many of these compounds are known to persist in ecosystems and have been associated with several diseases in humans [22]. Glyphosate, for example, has been linked with DNA damage and apoptosis in human cell lines [23–26]. For hence, the International Agency for Research on Cancer has classified this compound as a possible carcinogen [27]. Therefore, it is important to develop new strategies and biotechnological processes that could facilitate the control or elimination of this compound from wastewater and industrial sludges before its liberation to the environment resulting in the contamination of drinking water sources.

In this study, we present a novel strategy for the recovery of high-quality polyP through the uptake of glyphosate by a native *Bacillus cereus* strain. The release of inorganic phosphate (Pi) to the culture supernatant and the accumulation of intracellular polyP suggests that the *Bacillus cereus* strain designated as 6 P is capable to metabolize glyphosate on a Pi-insensitive manner.

## Materials and methods

### Soil sample collection

A soil sample from a glyphosate-exposed orange plantation site located in the north-eastern Mexican State of Nuevo León (25°57′57″ N 100°38′47″ W) was collected and stored in sterilized bags at 4 °C according to the Mexican environmental standard NMX-AA-132-SCFI-2006 (ref. 28). The site´s composite sample was sieved to eliminate roots and plant debris, and furtherly analyzed for humidity, pH, organic matter, exchangeable P, inorganic nitrogen and type of soil according to the Mexican environmental standard NOM-147-SEMARNAT/SSA1-2004 [29].

### Biomass acclimation and isolation of culturable bacterial strains

Biomass acclimation was carried out by adding 10 g of the site´s composite sample to 200 mL of LB broth supplemented with 100 ppm of the commercial glyphosate trade name Faena Fuerte^®^ 360. The batch bioreactor was incubated at 28 °C under aerobic conditions for 28 days, replenishing with fresh acclimation medium every seven days. At the end of the acclimation period, 10 mL aliquots of the microbial suspensions were washed with a sterile saline solution and preserved for further use in glycerol 25% at −20 °C.

The acclimatized-biomass was submitted to a serial dilution technique to isolate the culturable bacterial strains present in the consortium. The serial dilution was carried out in LB broth incubated at 28 °C and 150 rpm for 48 h, plating on LB agar and incubated at 28 °C for 3 days. Seven morphologically different colonies from the crowding on the surface of LB agar were selected and purified by repeated sub-culturing in LB media. The purified strains were preserved as described previously.

### Screening for polyphosphate accumulation by glyphosate-acclimatized bacterial isolates

The seven bacterial isolates were tested to evaluate their individual biological capacity to accumulate polyP as a direct effect of glyphosate uptake. These experiments were performed on minimal mineral medium (MMM), containing: MgSO_4_ · 7 H_2_O (0.2 g), KCl (0.2 g), CaCl_2_ · 2 H_2_O (0.001 g), Fe·NH_4_·Citrate (0.001 g), SL-4 trace elements solution (1 mL; [30]) and MEM vitamin solution (1 mL; Sigma-Aldrich, St. Louis, USA) in 1 L of distilled water. As a control, the MMM was supplemented with nutrients equivalent to Redfield´s ratio: sodium acetate 110 mM, NH_4_Cl 14 mM and NaH_2_PO_4_ 1 mM as carbon, nitrogen and phosphorus sources respectively. For bioassays, N-(phosphonomethyl)glycine 96% (Sigma-Aldrich, St. Louis, USA) was supplied as a source of phosphorus (1 mM) instead of NaH_2_PO_4_ used in the control.

All bioassays were performed in triplicate using 125 mL Erlenmeyer flasks containing 30 mL of experimental MMM adjusted to an initial 0.05 OD_620_ of each bacterial isolate. Experimental flasks were incubated at 28 °C and 150 rpm in a MaxQ 4000 orbital shaker (Thermo Fisher Scientific; Waltham, USA). End-point samples were collected after 120 h of experimentation and analyzed for *1)* microbial growth, quantified by the increase in total proteins following the Bradford assay [31] using a Cary 50 spectrophotometer (Agilent Technologies; Santa Clara, USA); *2)* glyphosate concentration in experimental supernatants, determined by the spectrophotometric method described by Waiman et al.; [32] *3)* release of inorganic phosphate to the culture supernatant during catabolism, assayed using the BioMol-Green reagent (ENZO Life Sciences; Farmingdale, USA) following the manufacturer´s protocol; *4)* monitoring of intracellular polyP granules, visualized by staining the cells with 500 μg/mL DAPI in 25 mM Tris-HCl at pH 7.0 as described by Kulakova et al. [33]. Stained cells were visualized at 1000x using a Leica DM-3000 fluorescent microscope with a DAPI filter cube (Leica Microsystems; Wetzlar, Germany); and *5)* monitoring of intracellular polyhydroxyalkanoate (PHA) granules as response to cellular nutritional stress, visualized by staining the cells with 30 μL Nile red 1% as described by Tan et al. [34] and observed at 1000x using fluorescence microscopy with a Texas Red filter cube.

### Extraction and characterization of intracellular polyphosphate

Intracellular polyP granules were extracted according to the methodology described by Kulakova et al. [33], in which the bacterial pellet attained from 10 mL of culture media was resuspended in 50 μL H_2_SO_4_ 1 M and incubated at room temperature for 5 min. This mix was neutralized with 50 μL NaOH 2 M and added 100 μL of Tris-HCl pH 7. The remaining mix was centrifuged at 13,000 r.p.m. for 5 min, and 600 μL NaI 6 M along with 600 μL of isopropyl alcohol were added to the supernatant. After final centrifugation at 13 000 rpm for 5 min, the polyP pellet was submitted to a Spectrum 100 FTIR spectrometer (PerkinElmer Inc; Waltham, USA), using 50 scans at 4 cm^−1^ resolution. The spectra were recorded in the region of 4000–650 cm^−1^ using a KBr window for solutions.

### Polyphosphate production yield and glyphosate biodegradation kinetics

The polyphosphate-accumulating bacterial isolate designated as 6 P was submitted to a kinetic study to evaluate its efficiency to biodegrade glyphosate along with its capacity to accumulate polyP. This bioassay was performed in triplicate using 125 mL Erlenmeyer flasks containing 30 mL of experimental MMM supplemented with N-(phosphonomethyl)glycine 96% as a phosphorus source (1 mM), adjusted to an initial 0.05 OD_620_ of the microbial suspension. Experimental flasks were incubated at 28 °C and 150 rpm for 240 h. Samples were collected every 24 h during the first 120 h, and after that, every 48 h until the completion of the experiment. Experimental samples were analyzed for the following: (1) microbial growth; (2) glyphosate concentration in experimental supernatants; and (3) release of inorganic phosphate to the culture supernatant during catabolism as described previously. Polyphosphate production yield was determined as dry weight of the intracellular polyP extracted according to Kulakova et al. [33] per litre of culture.

The glyphosate biodegradation rate constants were calculated using the modified Hockey-Stick model [35]. The following equations related to this method were used:

$${{C = C}}_{\mathrm{0}}\, {{{\rm{for}}:t}} \le {{t}}_{\mathrm{b}},$$

$${{C = C}}_{{0}}\, {{e}}^{{{ - k(t - tb)}}}\, {{{\rm{for}}:t > t}}_{\mathrm{b}},$$

where *C* is the concentration of glyphosate at time *t*, *C*_0_ is the concentration of glyphosate applied at time *t* *=* *0* in the experimental assay, *k* is the biodegradation rate constant of glyphosate and *t*_b_ is the breakpoint at the time at which rate constant changes and biodegradation start.

Glyphosate half-life (*T*_½_) was calculated according the following equation: [35]

$${{T}}_{{\raise0.5ex\hbox{$\scriptstyle 1$}\kern-0.1em/\kern-0.15em \lower0.25ex\hbox{$\scriptstyle 2$}}}{{ = t}}_{\mathrm{b}}{\mathrm{ + ln}}\,{{2/K}}.$$

### Identification of the polyphosphate-accumulating isolate 6P

The identity of the bacterial isolate designated as 6 P was confirmed by the 16s rRNA sequence alignment with the non-redundant nucleotide database held in the National Center for Biotechnology Information (NCBI), using the Basic Local Alignment Search Tool (nucleotide BLAST, http://blast.ncbi.nlm.nih.gov/Blast). To do so, DNA from the bacterial isolate was extracted using a PrepEase® Genomic DNA Isolation Kit (Thermo Fischer Scientific Inc; Waltham, USA) following the manufacturer´s protocol. DNA was resuspended in Tris 10 mM (pH 8.0) and quantified with a NanoDrop ND-1000 spectrophotometer (NanoDrop Technologies; Wilmington, USA). 1.6 μg/μl of the DNA template was used for the PCR amplification of the 16s rRNA gene, performed using the universal primers 27 F (5′-AGAGTTTGATCCTGGCTCAG-3′) y 132 R (5′-GGTTACCTTGTTACGACTT3′). The PCR reaction was carried out in a Mastercycler® pro thermocycler (Eppendorf AG; Hamburg, Germany) with the following program: 94 °C for 4 min, followed by 35 cycles of 92 °C for 1.5 min, 50 °C for 1.5 min, 72 °C for 2 min, and completing with 72 °C for 10 min. The purified PCR product was submitted to Macrogen Corp. (Rockville, USA) for sequencing.

To corroborate the molecular identity of the strain 6 P, the resulting 16s rRNA gene sequence was aligned against 14 representative members of the phylum Firmicutes (Table 1) using ClustalW. The phylogenetic tree was computed by the Minimum Evolution method using the MEGA 7 software with 1 000 bootstrap repetitions [36, 37].

**Table 1:** 16 s rRNA gene sequences of representative members of the Firmicutes phylum used in the phylogenetic analysis

Microorganism	Strain code	Accession number
*Related members of the genus Bacillus*		
*B. cereus*	ATCC 14579	AF290547.1
	ATCC 27877	Z84581.1
	ATCC 10987	AJ577290.1
	ATCC 4342	AJ577288.1
*B. subtilis*	CCM 1999	DQ207730.2
*B. licheniformis*	B-6-4J	D31739.1
*B. anthracis*	ATCC 10340	AB190217.1
*B. clausii*	DSM 8716	X76440.1
*B. megaterium*	C1	AJ491841.1
*Related members of the phylum Firmicutes*		
*Lactobacillus gallinarum*	ATCC 3319	AJ242968.1
*Lactobacillus amylotrophicus*	NRRL B-4435	AM236150.1
*Clostridium argentinense*	ATCC 27322	NR_029232.1
*Clostridium tertium*	ATCC 14573	AJ245413.1
*Staphylococcus aureus*	ATCC 25923	U02910.1

## Results and discussions

### Screening for polyphosphate accumulation by glyphosate-acclimatized bacterial isolates

**Fig. 1:**
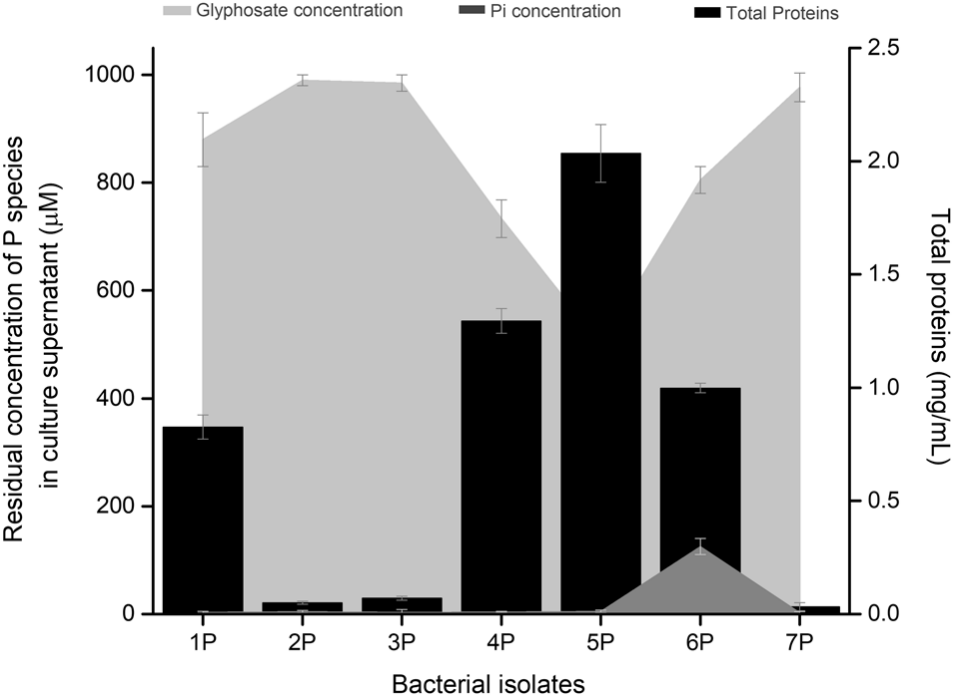
Profiles of microbial growth and concentration of phosphorus species (glyphosate and inorganic phosphate) in the culture supernatant after 120 h of experimentation by bacterial isolates grown on N-(phosphonomethyl)glycine 96% as a phosphorus source (1 mM). Error bar represents standard deviation (*n* = 3)

**Fig. 2:**
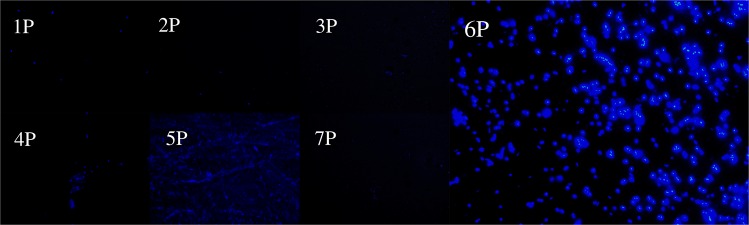
Fluorescence microscopic analysis for the evaluation of polyP accumulation by glyphosate-acclimatized bacterial isolates grown on 1 M N-(phosphonomethyl)glycine 96% as sole phosphorus source. Bacterial cells from strains 1 P, 2 P, 3 P, 4 P, 5 P, 6 P, and 7 P stained with DAPI after 120 h of incubation

Seven bacterial strains were isolated from a glyphosate-exposed orange plantation sample, which chemical characteristics are reported in Table 2. These isolates were exposed to 1 mM *N*-(phosphonomethyl)glycine supplied as phosphorus source and exhibited different patterns of growth and glyphosate consumption (Fig. 1). While strains 1 P, 4 P, 5 P, and 6 P could be considered as potential candidates for the development of a glyphosate biodegradation process due to the significant increase of total proteins and the decrease of glyphosate concentration in the culture supernatant, only the strain 6 P was able to release Pi to the supernatant (Fig. 1) and accumulate polyP intracellularly (Fig. 2) after 120 h of experimentation.

**Table 2:** Chemical characteristics of the glyphosate-exposed orange plantation sample

Location	pH	Humidity (%)	Organic matter (%)	Exchangeable P (ppm)	Inorganic nitrogen (%)	Type of soil
25°57′57″N	7.75	19.46	1.21	22.16	0.07	Vertisol (Clay 41%, Silt 53%, Sand 6%)
100°38′47″W

Glyphosate biodegradation by soil bacteria has been reported extensively since 1977 [38–43]. However, to date, there has not been reported a process of accumulation of polyP occurring in conjunction with the biodegradation of this herbicide. This is due because the cleavage of glyphosateʼs C–P bond has been demonstrated to be universally dependent on the concentration of inorganic phosphate present in the surrounding environment of the cell [44–48]. The in-vivo C–P lyase activity, enzyme responsible for the cleavage of glyphosateʼs C–P bond, has been confirmed to be both physiologically and genetically regulated as a member of the Pho regulon, which is expressed under phosphate limitation conditions [45, 49, 50]. While the concentration by which the Pho regulon may be activated varies between organisms, it has been accepted that inorganic phosphate concentrations below 4 μM leads to the activation of the two-component regulatory system PhoR-PhoB responsible for the expression of C–P lyase and other P-scavenging genes [51, 52]. Therefore, the biosynthesis of the C–P lyase is repressed by the Pi released during the degradation of the herbicide, which in turn, inhibits the complete mineralization of the phosphonate molecule and any potential accumulation of intra- or extracellular Pi [53].

**Fig. 3:**
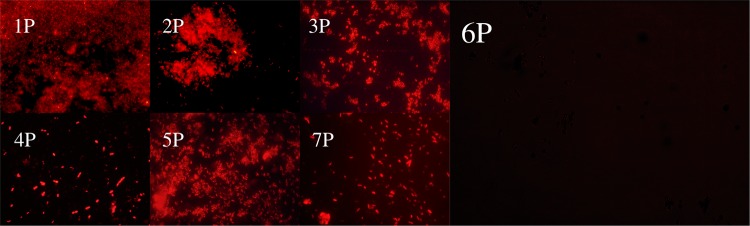
Fluorescence microscopic analysis for the evaluation of nutritional stress on glyphosate-acclimatized bacterial isolates grown on 1 M N-(phosphonomethyl)glycine 96% as sole phosphorus source. Bacterial cells from strains 1 P, 2 P, 3 P, 4 P, 5 P, 6 P, and 7 P stained with Nile red 1% after 120 h of incubation

Interestingly, the strain 6 P did not exhibit an accumulation of PHA granules in comparison with the rest of the studied isolates (Fig. 3). This is important because PHA has long been reported as a mechanism of survivability and adaptation against microbial stress [34]. As PHA tend to be accumulated under Pi-limitation (as well as other nutrients, such as nitrogen, oxygen, among others) [54], it can be inferred that this isolate was not under metabolic stress during the experiment. This suggests the strain 6 P, in contrast of the rest of the studied microorganisms, was able to obtain a continuous supply of Pi from *N*-(phosphonomethyl)glycine, which was supplied as the only available source of P. Moreover, the strain 6 P was able to accumulate the excess of Pi in the form of internal polyP granules, and even release part of it to the culture supernatant, as descrived previously.

### Extraction and characterization of intracellular polyphosphate

**Fig. 4:**
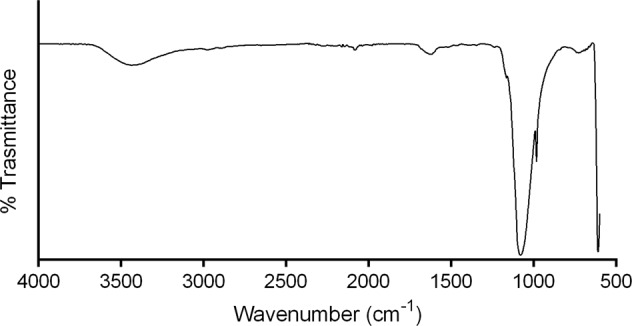
Fourier transform infra-red spectrum of purified polyphosphate extracted from the biomass of the bacterial isolate 6 P grown on 1 M N-(phosphonomethyl)glycine 96% as sole phosphorus source

As observed in Fig. 4, the FTIR spectrum of the biomaterial extracted from the biomass of the strain 6 P showed strong bands at 900 and 1100 cm^−1^. These have been associated with the P-O and P=O bonds present in the phosphate moiety of polyP [55]. The absence of additional bands in the spectrum suggests that no additional chemical elements or functional groups are present in the molecular structure of the analyzed material, discarding the presence of organophosphorus compounds [56]. Therefore, we concluded the identity of the biomaterial to be polyphosphate. The purity observed in the FTIR spectrum suggest that the strain 6 P can produce high-quality polyP and can be considered as a potential candidate for the generation of an innovative biotechnological process to produce this important biopolymer through the biodegradation of the herbicide glyphosate.

### Polyphosphate production yield and glyphosate biodegradation kinetics

**Fig. 5:**
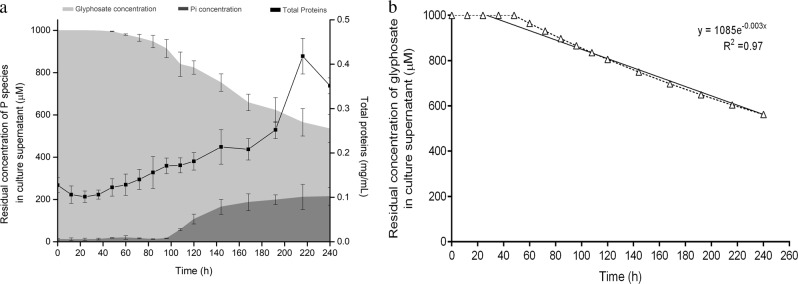
**a** Growth curve of the glyphosate-acclimatized bacterial isolate 6 P in MMM containing 1 M N-(phosphonomethyl)glycine 96% as sole phosphorus source, and changes in the concentration of phosphorus species (glyphosate and inorganic phosphate) in the culture supernatant at various stages of cellular growth. Error bar represents standard deviation (*n* = 3); (**b**) modeling of the glyphosate biodegradation kinetics by the modified Hockey–Stick mathematical model

A more detailed study on the glyphosate biodegradation kinetics by the strain 6 P demonstrated that Pi concentration remained undetected in the culture supernatant for the first 96 h of experimentation (Fig. 5a). According to the manufacturer, the BIOMOL^®^ Green method is capable of detecting Pi concentrations down to 0.5 μM, which coincide with the ideal condition for the induction of the C–P lyase [51, 52, 57]. However, after this period, Pi levels rose above the C–P lyase induction concentration until reaching nearly 20% of the initial glyphosate concentration at the end of the experimentation time. These Pi levels are known to have inhibited the C–P lyase activity of many bacterial species, including *Acidithiobacillus ferrooxidans* [58], *Trichodesmium erythraeum* [59], and *Escherichia coli* [45]. Despite this paradox, the continuous increase of total proteins observed along the experiment suggests that cells of the strain 6 P were biologically active, being able of utilizing glyphosate as a source of P for microbial growth in a Pi-insensitive manner. Moreover, the strain 6 P was able to accumulate part of the excess Pi as PolyP granules, which were visualized only after 120 h of experimentation, reaching a final production yield of 4 mg l^−1^.

Pi-insensitive degradation of glyphosate is a rare phenomenon. It was first reported in *Alcaligenes* sp. GL. This bacterial strain exhibited a base level of glyphosate degrading activity during growth in the presence of Pi or in complex media. However, the complete removal of Pi from the medium caused a drastic stimulation of glyphosate uptake [60]. On the other hand, the mutant *Arthrobacter* sp. GLP-1/Nit-1 was capable to excrete 90% of the glyphosate-derived phosphorus when this phosphonate was supplied as nitrogen and phosphorus source. Nevertheless, the native strain *Arthrobacter* sp. GLP-1 was able to utilize glyphosate only in the absence of Pi [61]. To date, a true Pi-independency has only been reported on the biodegradation of biogenic phosphonates by environmental strains that possess analog enzymes of phosphonatase [62], phosphonoacetate hydrolase [63, 64], and phosphonopyruate hydrolase [65, 66]. Therefore, the Pi-insensitive degradation of glyphosate by *B. cereus* 6 P is noteworthy and represent a feasible biotechnological process to mineralize glyphosate under environmental conditions.

The biodegradation of glyphosate was adjusted to a modified Hockey–Stick first-order kinetic model (Fig. 5b). This mathematical equation, which is commonly used for the calculation of pesticides biodegradation rates [35], describes the behavior of a pesticide where the initial concentration is not constant but declines very slowly up to a breakpoint (t_b_). As shown in Fig. 5a, this breakpoint was observed at 48 h of experimentation, when glyphosate concentration began to decrease faster, reaching a final concentration of 623 μM. The kinetic constant was calculated on 0.003 h^−1^ and the glyphosate half-life on 279 h.

### Identification of the polyphosphate-accumulating isolate 6P

**Fig. 6:**
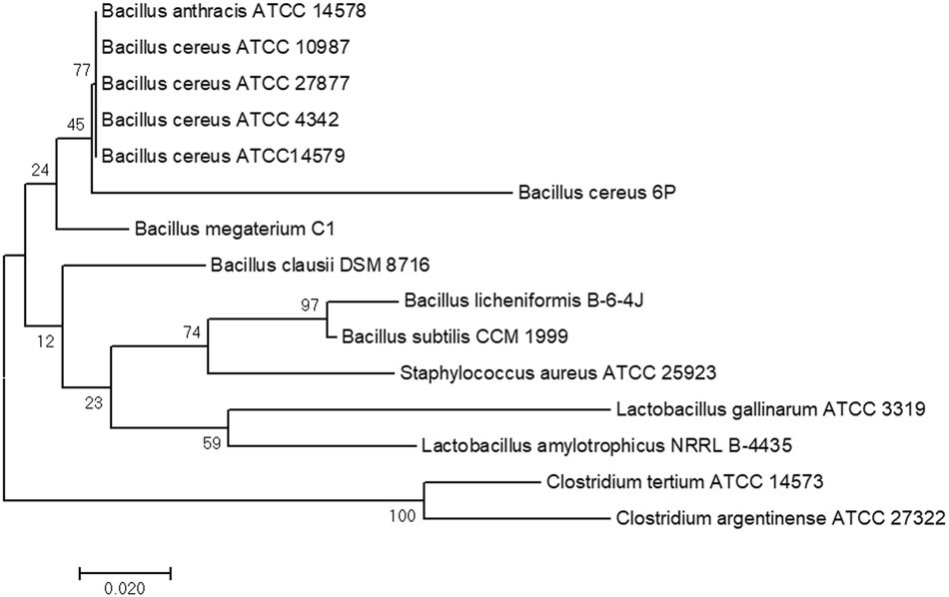
Phylogenetic tree showing the relationship between *Bacillus cereus* 6 P and 14 representatives of the Firmicutes phylum as inferred by the Minimum Evolution method. The percentage of replicate trees in which the associated taxa clustered together in the bootstrap test (1000 replicates) are shown next to the branches

The resulting nucleotide sequence established that the strain 6 P belonged to the *Bacillus cereus* species with a 97% identity based on the BLASTN analysis. When compared with other members of the Firmicutes phylum, the strain 6 P clustered along with other *B. cereus* species, confirming the identity of this native bacterial isolate (Fig. 6). This result is consistent with the natural occurrence and distribution of the species, since the cells and spores of *B. cereus* are present in a wide variety of environmental niches, mainly as components of the rhizomicrobiota present in soil, in endophytic communities of growing plants, or as potential pathogens of insects and mammals [67]. Having been isolated from different soils dedicated to agriculture [68, 69], the presence of a strain of *B. cereus* in an orange plantation site may be expected.

On the other hand, the biological capability to accumulate polyP is widely distributed among Firmicutes, since these commonly synthesize it to attain certain ecological advantages [70]. This molecule, rich in high-energy phosphoanhydride bonds, is known to act not only as a phosphate and energy storage material, but also as a key player in several physiological processes, such as flagellar mobility, biofilm development, quorum sensing and virulence [71]. By regulating the mechanism of response to stresses and stringencies, PolyP molecule increases the survivability of these microorganisms under unfavorable growth conditions [72]. Moreover, in *B. cereus*, the role of polyP has also been associated with the sporulation process, with polyP levels dropping rapidly when spore formation begins [73]. This suggests that the strain 6 P may be able to metabolize the glyphosate present in agricultural soils to provide the cell with a phosphate and energy storage material that could be used later in the sporulation process, assuring its long-term survival when faced with unfavorable growth conditions.

In conclusion, we have shown that the native bacterial isolate, designated as *Bacillus cereus* 6 P, is capable of accumulating intracellular polyP in response to the uptake of glyphosate as a source of P. As this process occurred independently of the Pi present in the culture supernatant, it can be suggested that *B. cereus* 6 P is currently the only bacterial strain capable of metabolizing glyphosate in a Pi-insensitive manner. In ecological terms, this would allow *B. cereus* 6 P and related species to accumulate PolyP in sites exposed to glyphosate before members of other species and phyla. Being directly related to the sporulation process, this new biological process would help ensure the long-term survival of this bacterial strain. On the other hand, the reaction conditions described in this work would overcome the existing biological limitations for the development of an efficient biotechnological process capable of mineralizing this herbicide. However, more work is required to determine its potential application for phosphorus recovery as part of the strategy for future food security, and all the ecological implications of this new process represents for the bacteria that are usually present in soils rich in glyphosate.
